# MicroRNA-181a–2–3p shuttled by mesenchymal stem cell-secreted extracellular vesicles inhibits oxidative stress in Parkinson’s disease by inhibiting EGR1 and NOX4

**DOI:** 10.1038/s41420-022-00823-x

**Published:** 2022-01-24

**Authors:** Jianjun Ma, Xiaoxue Shi, Mingjian Li, Siyuan Chen, Qi Gu, Jinhua Zheng, Dongsheng Li, Shaopu Wu, Hongqi Yang, Xue Li

**Affiliations:** 1 Department of Neurology, Henan Provincial People’s Hospital, Zhengzhou, 450003 P. R. China; 2 Department of Neurology, Zhengzhou University People’s Hospital, Zhengzhou, 450003 P. R. China; 3 Department of Neurology, Henan University People’s Hospital, Zhengzhou, 450003 P. R. China

**Keywords:** Biotechnology, Diseases

## Abstract

The current study investigated the physiological mechanisms by which extracellular vesicle (EV)-encapsulated miR-181a–2–3p derived from mesenchymal stem cells (MSCs) might mediate oxidative stress (OS) in Parkinson’s disease (PD). First, 6-hydroxydopamine (6-OHDA)-induced PD cell and mouse models were established, after which miR-181a–2–3p, EGR1, and NOX4 expression patterns were determined in SH-SY5Y cells and substantia nigra (SN) of PD mice. Next, the binding affinity among miR-181a–2–3p, EGR1, and NOX4 was identified using multiple assays. Gain- or loss-of-function experiments were further adopted to detect SH-SY5Y cell proliferation and apoptosis and to measure the levels of SOD, MDA, and ROS. Finally, the effects of miR-181a–2–3p from MSC-derived EVs in PD mouse models were also explored. It was found that miR-181a–2–3p was poorly expressed in 6-OHDA-induced SH-SY5Y cells, whereas miR-181a–2–3p from MSCs could be transferred into SH-SY5Y cells via EVs. In addition, miR-181a–2–3p could target and inhibit EGR1, which promoted the expression of NOX4. The aforementioned miR-181a–2–3p shuttled by MSC-derived EVs facilitated SH-SY5Y proliferation and SOD levels, but suppressed apoptosis and MDA and ROS levels by regulating EGR1 via inhibition of NOX4/p38 MAPK, so as to repress OS of PD. Furthermore, in PD mice, miR-181a–2–3p was carried by EVs from MSCs to alleviate apoptosis of dopamine neurons and OS, accompanied by increased expressions of α-syn and decreased 4-HNE in SN tissues. Collectively, our findings revealed that MSC-derived EV-loaded miR-181a–2–3p downregulated EGR1 to inhibit OS via the NOX4/p38 MAPK axis in PD.

## Introduction

Parkinson’s disease (PD), one of the leading chronic degenerative neurological forms of dyskinesia, poses a significant threat to the health of elderly populations [1]. PD is characterized by a plethora of cellular abnormalities, including oxidative stress (OS), proteasome stress, and mitochondrial dysfunction [2]. More importantly, OS is well-established to function as a key factor for cell dysfunction and death in PD [3], and further serves as the imbalance between reactive oxygen species and the ability of cells to produce an effective antioxidant response [4]. The last decade has also witnessed the discovery of the therapeutic potential of mesenchymal stem cells (MSC) in neurodegenerative disorders, including PD [5]. In a similar light, MSCs possess the ability to transfer specific types of extracellular vesicles (EVs) to achieve therapeutic effects [6]. More specifically, MSC–EVs have been previously highlighted as a potential target for treating PD [7], however, the specific mechanisms of MSC-derived EVs underlying in OS in PD remain to be further elucidated.

Inherently, EVs are described as membrane-derived vesicles, secreted by different types of cells under normal and pathological conditions [8], and further confer important roles in the pathophysiological processes of diseases, such as cancer and neurodegenerative disease [9]. In addition, EVs have been elucidated to function as important cell–cell communication entities in several physiological and pathological processes, acting as a carrier of various biomolecules, including microRNAs (miRNAs) [10]. These miRNAs are highly conserved, small noncoding RNAs with 21–23 nucleotides in length, and are capable of regulating relevant genes by binding to their 3′UTR [11]. Moreover, various researchers have documented the effects of miRNAs on PD [12]. Liu et al. have also confirmed that one such miRNA, namely microRNA-181a (miR-181a), was implicated in the progression of PD [13].

Existing research further suggests that miR-181a-5p can target early growth-response-1 (EGR1) transcription factor [14]. More specifically, EGR1, a prototypic Cys2–His2-type zinc-finger transcription factor, is known to be rapidly stimulated by various stimuli, such as growth factors, pro-inflammatory cytokines, etc. [15]. Interestingly, a prior study further indicated toward the potential roles of EGR1 in neuroinflammation in PD [16]. Furthermore, EGR1 is established as a transcriptional activator of the NOX4 protein in oxidative stress of diabetic kidney disease [17]. In addition, previous literature suggests that NOX4 also functions as a regulator in the development of PD [18]. Moreover, NADPH-oxidase catalytic subunits, including NOX4, have been reported in the brain of patients [18]. Meanwhile, the gain function of NOX4 was previously elucidated to be conducive to the activation of p38 mitogen-activated kinase (MAPK) pathway [19]. Unsurprisingly, the activated MAPK signaling like p38 has been documented in the pathogenesis of PD [20]. However, the mechanism by which MSC-derived EV communication affects OS in PD involving the interplay among miR-181a-5p, EGR1, and NOX4, remains elusive. Accordingly, we hypothesized that transfer of miR-181a-5p via MSC-derived EVs might alter OS in PD, and performed a series of experiments to validate our hypothesis.

## Results

### MiR-181a–2–3p overexpression inhibits 6-OHDA-induced OS injury in SH-SY5Y cells

**Fig. 1:**
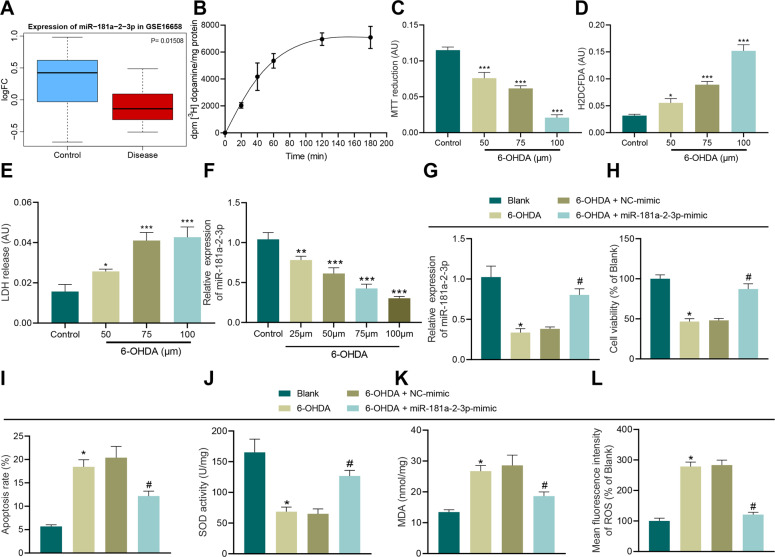
miR-181a–2–3p inhibits apoptosis and OS of SH-SY5Y cells. **A** The box plot of miR-181a–2–3p in GSE16658, the blue box on the left indicated expression of normal samples, and the red box on the right indicates miR-181a–2–3p expression in PD patients. **B** Radioactive dopamine-uptake test. **C** Cell viability in SH-SY5Y cells treated with 6-OHDA determined with MTT assay. **D** Intracellular oxidation level evaluated after treatment with different drug concentrations. **E** LDH-release assay. **F** miR-181a-2-3p expression detected in SH-SY5Y cells treated with 6-OHDA for 24 h. **G** miR-181a–2–3p expression detected in 6-OHDA-induced SH-SY5Y cells using RT-qPCR. **H** SH-SY5Y cell proliferation detected using CCK-8 assay after 6-OHDA treatment. **I** Apoptosis of SH-SY5Y cells treated with 6-OHDA detected using flow cytometry. **J** SOD level in SH-SY5Y cells treated with 6-OHDA detected using SOD kit. **K** MDA level in SH-SY5Y cells treated with 6-OHDA detected using MDA kit. **L** ROS levels in SH-SY5Y cells treated with 6-OHDA detected using DCFH-DA fluorescent staining. *p < 0.0*5*, **p < 0.01, ***p < 0.001 vs. control or SH-SY5Y cells without treatment. Cell experiments were repeated three times independently.

Initial analyses of the PD miRNA expression dataset GSE16658 from the Gene Expression Omnibus (GEO) database suggested that miR-181a-2-3p was downregulated in PD (Fig. 1A). In addition, the results of radioactive dopamine uptake, 3-(4,5-dimethyl-2-thiazyl)-2,5-diphenyl-2H-tetrazolium bromide (MTT), intracellular oxidation, and lactate-dehydrogenase (LDH) release assays, illustrated that cell viability was decreased, while oxidative pressure and LDH release were both gradually increased in a 6-OHDA-dependent manner, validating that PD model in vitro was successfully established (Fig. 1B–E). Moreover, we observed that miR-181a–2–3p expression in SH-SY5Y cells exhibited a decline after 6-OHDA induction in a concentration-dependent manner. Meanwhile, treatment with 100 μM 6-OHDA brought about the most profound inhibitory effect, and was thus selected to induce PD in vitro cell models in SH-SY5Y cells (Fig. 1F).

Additionally, the results of reverse-transcription quantitative polymerase-chain reaction (RT-qPCR) demonstrated that miR-181a–2–3p expression levels were decreased in 6-OHDA-treated SH-SY5Y cells, while being increased in cells transduced with miR-181a–2–3p mimic, verifying the successful transduction efficiency (Fig. 1G). As illustrated by cell-counting kit (CCK)-8 and flow cytometry, cell viability was decreased and apoptosis was increased in 6-OHDA-treated SH-SY5Y cells, while the opposite trends were observed in cells transduced with miR-181a–2–3p mimic (Fig. 1H, I). In addition, superoxide dismutase (SOD), malondialdehyde (MDA) kits, and DCFH-DA fluorescent staining were adopted to detect cell SOD, MDA, and ROS levels, respectively, which revealed that after 6-OHDA induction, SH-SY5Y cells presented with decreased SOD levels and elevated levels of MDA and ROS, while over-expressing miR-181a–2–3p brought about the opposite effects in SH-SY5Y cells (Fig. 1J–L). Collectively, these findings indicated that overexpression of miR-181a–2–3p inhibited 6-OHDA-induced OS injury in SH-SY5Y cells

### MSC–EVs deliver MiR-181a–2–3p to SH-SY5Y cells

**Fig. 2:**
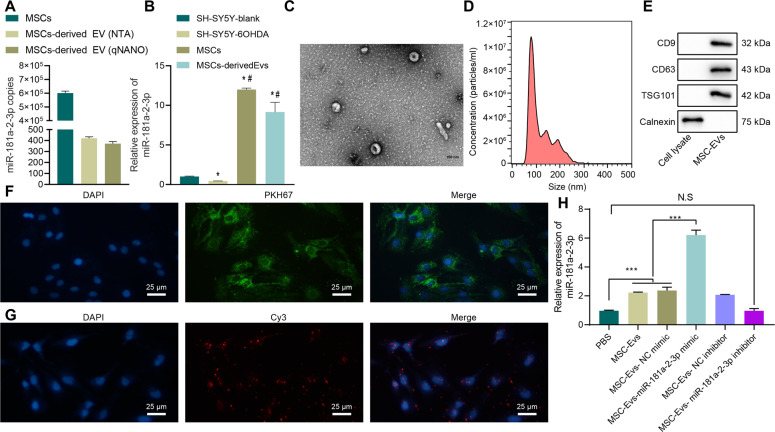
miR-181a–2–3p could be carried to SH-SY5Y cells through MSC–EVs. **A** Abundance of miR-181a–2–3p in MSCs and MSC-derived EVs. **B** miR-181a–2–3p expression by RT-qPCR. **C** EVs observed under the TEM. **D** EV diameter detected using dynamic light scattering. **E** EV surface markers detected using Western blot. **F** Uptake of MSC–EV by SH-SY5Y cells detected using EV PKH67 tracing method. **G** Cy3-labeled miR-181a–2–3p entered SH-SY5Y cells via MSC–EV (200×). **H** miR-181a–2–3p expression in the SH-SY5Y cells. *p < 0.05 vs. SH-SY5Y blank, ***p < 0.001, NS insignificant difference. Cell experiments were repeated three times independently.

The EVmiRNA online database suggested that miR-181a–2–3p was relatively enriched in MSCs compared with other cells (Supplementary Fig. 1). Subsequently, we performed an absolute quantitative analysis of miR-181a in MSCs and MSC–EVs, and confirmed that miR-181a was indeed enriched in MSCs and MSC–EVs (Fig. 2A). Moreover, we found that miR-181a–2–3p was upregulated in MSCs and MSC–EVs compared with SH-SY5Y blank or SH-SY5Y–6-OHDA (Fig. 2B). It is also noteworthy that MSC–EVs have been previously suggested to serve as a potential target for treating PD [7]. As a result, we hypothesized whether the miR-181a–2–3p carried by EVs might be a new regulatory miRNA for PD. In order to test the said hypothesis, we separated and observed MSC–EVs under a transmission electron microscope (TEM) (Fig. 2C), which illustrated that MSC–EVs were round or elliptical membranous vesicles with basically the same morphology, with the size of EVs measured varying from 30 nm to 200 nm (Fig. 2D). In addition, the results of Western blot assay demonstrated that contents of surface markers CD9, CD63, and TSG101 were highly expressed in EVs, while calnexin was not expressed, which confirmed the successful extraction of EVs (Fig. 2E).

In order to study whether the MSC–EVs could carry miR-181a–2–3p to SH-SY5Y cells, we cocultivated PKH67 (green)-labeled EVs with SH-SY5Y cells for 24 h, and observed the uptake of EVs by NP cells, and it was found that SH-SY5Y cells had obviously taken up PKH67 EVs (Fig. 2F). Furthermore, we found that miR-181a–2–3p was expressed in SH-SY5Y cells, indicating that MSC–EV could carry miR-181a–2–3p into SH-SY5Y cells (Fig. 2G). The results of RT-qPCR further displayed that when cocultured with MSC–EV, miR-181a–2–3p expression levels were elevated in SH-SY5Y cells (Fig. 2H). Together, these findings suggested that MSC–EVs could deliver miR-181a–2–3p to SH-SY5Y cells.

### MSC–EVs carrying MiR-181a–2–3p inhibit 6-OHDA-induced OS injury

**Fig. 3:**
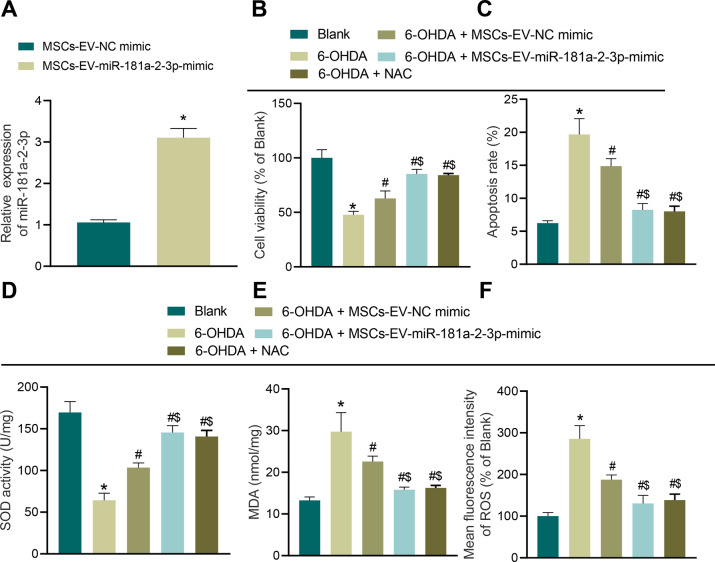
MSC–EVs carrying miR-181a–2–3p inhibit apoptosis and OS of SH-SY5Y cells. **A** miR-181a–2–3p expression determined by RT-qPCR. **B** Cell proliferation in SH-SY5Y cells co-incubated with MSC–EVs. **C** Apoptosis of SH-SY5Y cells co-incubated with MSC–EVs. **D** SOD level in SH-SY5Y cells co-incubated with MSC–EVs using SOD kit. **E** MDA level in SH-SY5Y cells co-incubated with MSC–EVs using MDA kit. **F** ROS levels in SH-SY5Y cells co-incubated with MSC-EVs using DCFH-DA fluorescent staining. *p < 0.05 vs. MSC–EV–NC mimic or SH-SY5Y cells without treatment, ^#^*p* < 0.05 vs. 6-OHDA-induced SH-SY5Y cells; ^$^*p* < 0.05 vs. SH-SY5Y cells co-incubated with MSC–EV–NC mimic. Cell experiments were repeated three times independently.

To further determine whether EV-encapsulated miRNA confers potential therapeutic effect on OS injury in SH-SY5Y cells, we overexpressed miR-181a–2–3p in MSCs, and extracted EVs to detect its expression. It was observed that miR-181a–2–3p expression levels were elevated in SH-SY5Y cells transduced with MSC-EV-miR-181a–2–3p mimic (Fig. 3A). Subsequently, MSC–EVs were cocultured with SH-SY5Y cells, and it was found that MSC–EVs, MSC–EV mimic, or antioxidant N-acetyl-l-cysteine (NAC) treatment promoted cell viability and inhibited apoptosis in 6-OHDA-treated cells, meanwhile, MSC–EVs carrying miR-181a–2–3p enhanced cell viability and evidently suppressed apoptosis (Fig. 3B, C). Moreover, the results of SOD detection, MDA detection, and DCFH-DA fluorescence staining illustrated that MSC–EVs, MSC–EV mimic, or NAC treatment elevated SOD levels and reduced the levels of MDA and ROS, while MSC–EVs carrying miR-181a–2–3p were found to elevate SOD levels and significantly decrease the levels of MDA and ROS (Fig. 3D–F). Additionally, we also documented similar findings in mouse primary neuron cells (Supplementary Fig. 2A–F). Taken together, these findings indicated that MSC–EVs containing miR-181a–2–3p inhibit 6-OHDA-induced OS injury in neuroblastoma cells and neuron cells.

### MiR-181a–2–3p targets EGR1

**Fig. 4:**
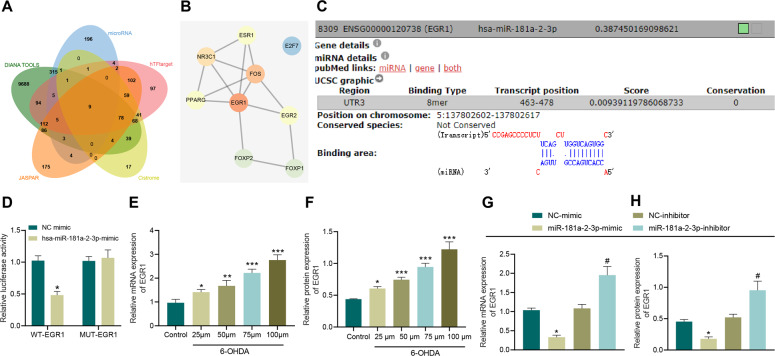
miR-181a–2–3p targets EGR1. **A** The downstream genes of miR-181a–2–3p predicted using DIANA TOOLS and mircroRNA and Venn map of the human transcription factor obtained from hTFtarget, Cistrome and JASPAR. **B** The interaction network of proteins of 9 important transcription factors constructed by String. Red represents the higher core degree, and blue represents the lower core degree. **C** The binding sites between miR-181a–2–3p and EGR1 predicted using DIANA TOOL. **D** The binding of miR-181a–2–3p to EGR1 using dual-luciferase assay. **E** EGR1 mRNA level in SH-SY5Y cells treated with 6-OHDA determined using RT-qPCR. **F** Western blot analysis of EGR1 protein level in SH-SY5Y cells treated with 6-OHDA. **G** EGR1 mRNA level in SH-SY5Y cells treated with miR-181a–2–3p mimic or inhibitor determined using RT-qPCR. **H** Western blot analysis of EGR1 protein level in SH-SY5Y cells treated with miR-181a-2-3p mimic or inhibitor. *p < 0.05 vs. NC mimic or *p < 0.0*5*, **p < 0.01, ***p < 0.001 vs. SH-SY5Y cells without treatment; ^#^*p* < 0.05 vs. NC inhibitor. Cell experiments were repeated three times independently.

DIANA TOOLS and microRNA databases predicted a total of 10508 and 546 downstream genes of miR-181a–2–3p, respectively. Additionally, 678, 318, and 639 human transcription factors were obtained through the hTFtarget, Cistrome and JASPAR databases, respectively. Subsequently, 9 important downstream transcription factors of miR-181a–2–3p were found at the intersection of the aforementioned factors (Fig. 4A). A 9-transcription-factor protein mutual rental network was constructed using the String database, and EGR1 was found as the transcription factor with the highest core level (Fig. 4B), and might serve as the key downstream transcription factor of miR-181a–2–3p. DIANA TOOLS were retrieved another time to obtain the binding site between miR-181a–2–3p and EGR1 (Fig. 4C). As reflected by the results of dual-luciferase reporter gene assay, the luciferase activity of EGR1-WT was inhibited by miR-181a–2–3p mimic (Fig. 4D). Moreover, RT-qPCR and Western blot analysis demonstrated that following treatment with 6-OHDA, EGR1 expression levels were increased in a 6-OHDA concentration-dependent manner (Fig. 4E, F, and Supplementary Fig. 3A). In addition, miR-181a–2–3p inhibited the EGR1 levels, whereas inhibition of miR-181a–2–3p brought about the opposite results (Fig. 4G, H, and Supplementary Fig. 3B).

### MiR-181a–2–3p inhibits OS injury by targeting EGR1

**Fig. 5:**
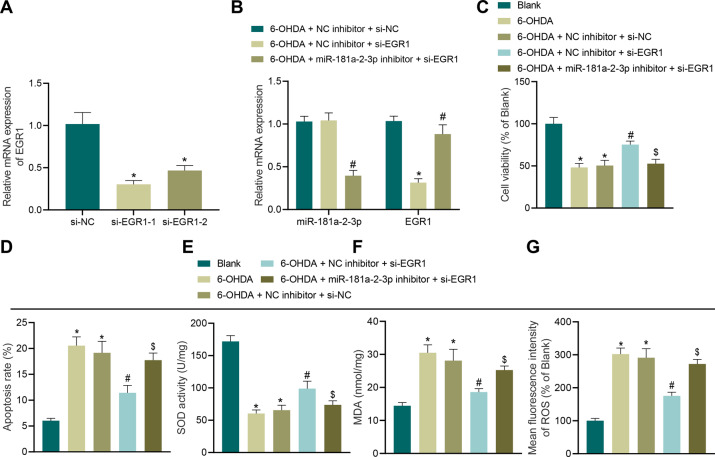
miR-181a–2–3p suppresses apoptosis and OS of SH-SY5Y cells by inhibiting EGR1 expression. **A** The knockdown effect of the two interference sequences of EGR1. **B** Expression of miR-181a–2–3p and EGR1 in SH-SY5Y cells after different treatments. **C** Cell proliferation in SH-SY5Y cells after different treatments. **D** Apoptosis of SH-SY5Y cells after different treatments. **E** SOD level in SH-SY5Y cells after different treatments detected using SOD kit. **F** MDA level in SH-SY5Y cells after different treatments using MDA kit. **G** ROS levels in SH-SY5Y cells after different treatments detected using DCFH-DA fluorescent staining. *p < 0.05 vs. si-NC, NC inhibitor + si-NC, or cells without treatment, ^#^*p* < 0.05 vs. NC inhibitor + si-EGR1 or NC inhibitor + si-NC; ^$^*p* < 0.05 vs. NC inhibitor + si-EGR1. Cell experiments were repeated three times independently.

Furthermore, we established two interference sequences of EGR1, and the results of RT-qPCR illustrated that the two interference sequences of EGR1 could inhibit the EGR1 expression (Fig. 5A), and siRNA (si)-EGR1–1 was selected for follow-up experiments. In addition, RT-qPCR results illustrated that siRNA targeting EGR1 reduced the EGR1 expression, whereas siRNA targeting EGR1 combining with miR-181a–2–3p inhibitor decreased the miR-181a–2–3p levels and increased those of EGR1 (Fig. 5B). Relative to untreated cells, cells showed inhibited cell viability and promoted apoptosis. Meanwhile, compared with negative-control (NC) inhibitor + si-NC, cell viability was increased and apoptosis was reduced in cells transduced with si-EGR1 alone, while further transduction of miR-181a–2–3p inhibitor resulted in diminished cell viability and promoted apoptosis (Fig. 5C, D). Additionally, the results of SOD detection, MDA detection and DCFH-DA fluorescent staining displayed that compared with cells without treatment, 6-OHDA-induced SH-SY5Y cells presented with decreased SOD levels and elevated levels of MDA and ROS. On the other hand, increased SOD levels and decreased levels of MDA and ROS were observed in cells transduced with si-EGR1 alone, while additional transduction of miR-181a–2–3p inhibitor brought about diminished SOD levels and increased levels of MDA and ROS (Fig. 5E–G). Altogether, these findings suggested that miR-181a–2–3p attenuated OS injury by targeting EGR1.

### EGR1 promotes OS injury by upregulating NOX4

**Fig. 6:**
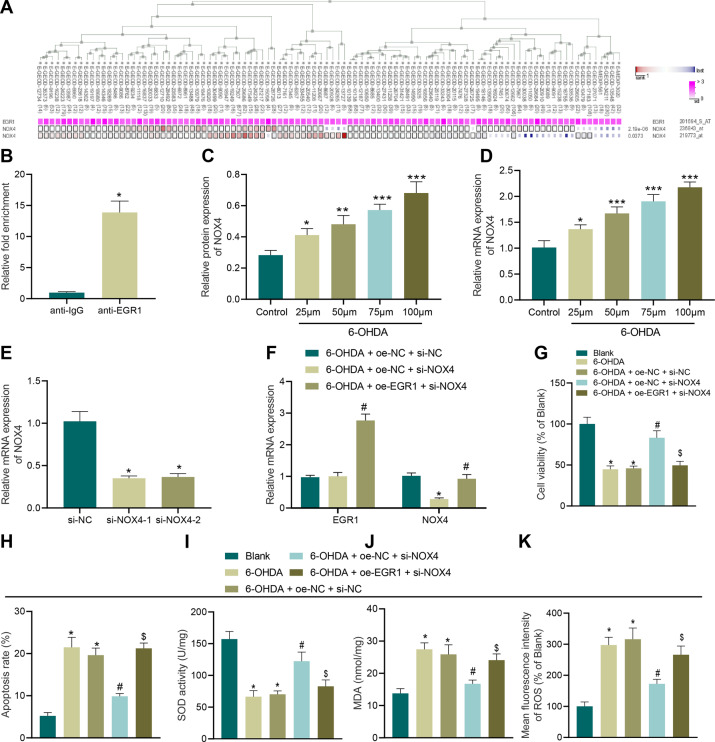
EGR1 promotes apoptosis and OS of SH-SY5Y cells by regulating NOX4. **A** Significant co-expression of EGR1 and NOX4 analyzed using MEM. **B** EGR1 enriched in the NOX4 promoter region verified by ChIP assay. **C** NOX4 mRNA level in SH-SY5Y cells treated with 6-OHDA at different concentrations detected using RT-qPCR. **D** NOX4 protein level in SH-SY5Y cells treated with 6-OHDA at different concentrations. **E** The knockdown effect of the two interference sequences of NOX4. **F** Expression of EGR1 and NOX4 in SH-SY5Y cells after different treatments measured using RT-qPCR. **G** Cell proliferation in SH-SY5Y cells after different treatments. **H** Cell apoptosis in SH-SY5Y cells after different treatments. **I** SOD level in SH-SY5Y cells after different treatments. **J** MDA level in SH-SY5Y cells after different treatments. **K** ROS levels in SH-SY5Y cells after different treatments detected using DCFH-DA fluorescent staining. *p < 0.05 vs. si-NC, oe-NC + si-NC, or SH-SY5Y cells without treatment, ^#^*p* < 0.05 vs. oe-NC + si-NOX4, oe-NC + si-NC; ^$^*p* < 0.05 vs. oe-NC + si-NOX4. Cell experiments were repeated three times independently.

MEM analysis was carried out to investigate the presence of a significant co-expression relationship between EGR1 and NOX4 (Fig. 6A). EGR1 was found to be significantly enriched in the NOX4 promoter (Fig. 6B). Subsequently, SH-SY5Y cells were treated with 6-OHDA at different concentrations for 24 h, and the expression patterns of NOX4 were determined by RT-qPCR and Western blot analysis. It was observed that NOX4 expression levels were increased after 6-OHDA treatment, and increased in a 6-OHDA concentration-dependent manner (Fig. 6C, D).

In order to further explore the function of NOX4 in PD, we established two interference sequences of NOX4. Both interference sequences were validated to inhibit the NOX4 expression (Fig. 6E), and si-NOX4–1 with more profound interfering efficiency was selected for subsequent experiments. As demonstrated by the results of RT-qPCR, siRNA-targeting NOX4 reduced NOX4 expression levels, and upregulated EGR1, whereas depletion of NOX4 elevated EGR1 expression and elevated NOX4 expression levels in cells (Fig. 6F). In addition, CCK-8 and flow cytometry results illustrated that compared with cells without treatment, cell viability was inhibited and apoptosis was enhanced in 6-OHDA-treated cells. Moreover, facilitated cell viability and repressed apoptosis were observed after transduction with si-NOX4 alone, while these trends were reversed by further injection of oe-EGR1 (Fig. 6G–H). The results of SOD detection, MDA detection, and DCFH-DA fluorescent staining displayed that compared with untreated cells, SOD levels were decreased and MDA and ROS levels were elevated in 6-OHDA-induced cells. On the other hand, increased SOD levels decreased and decreased levels of MDA and ROS were found after transduction with si-NOX4 alone, while these trends were reversed by further injection of oe-EGR1 (Fig. 6I–K).

### NOX4 promotes OS injury by activating p38 MAPK pathway

**Fig. 7:**
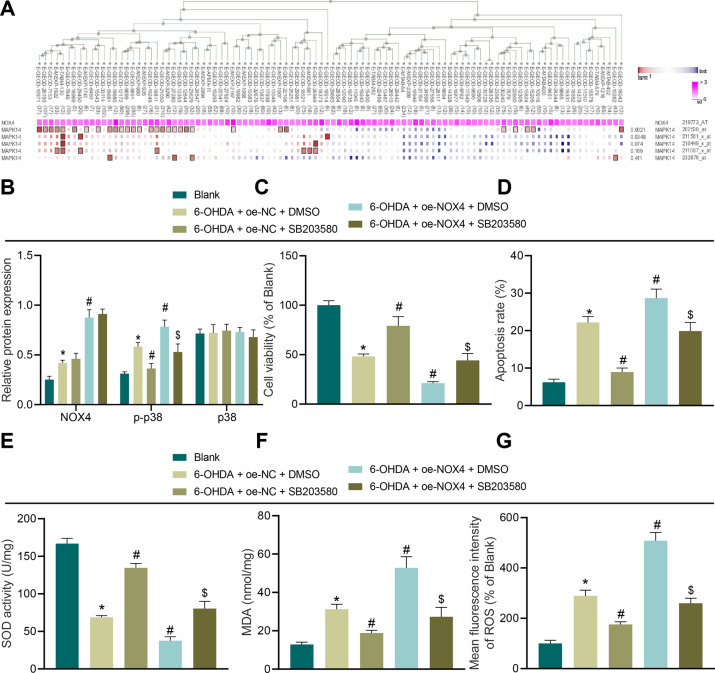
p38 MAPK pathway mediates NOX4 to promote apoptosis and OS of SH-SY5Y cells. **A** MEM analysis of NOX4 and p38 MAPK significant co-expression. **B** The levels of NOX4, p–p38, and p38 in SH-SY5Y cells after different treatments measured by Western blot assay. **C** Cell proliferation in SH-SY5Y cells after different treatments. **D** Cell apoptosis in SH-SY5Y cells after different treatments. **E** SOD level in SH-SY5Y cells after different treatments. **F** MDA level in SH-SY5Y cells after different treatments. **G** ROS levels in SH-SY5Y cells after different treatments detected using DCFH-DA fluorescent staining. *p < 0.05 vs. SH-SY5Y cells without treatment, ^#^*p* < 0.05 vs. oe-NC + DMSO; ^$^*p* < 0.05 vs. oe-NOX4 + DMSO. Cell experiments were repeated three times independently.

NOX4 and p38 MAPK (NCBI-included name: MAPK14) were co-expressed (Fig. 7A). To further verify the effect of the p38 MAPK signaling pathway on SH-SY5Y cell OS injury, we treated SH-SY5Y cells with the p38 MAPK inhibitor SB203580. Subsequent findings revealed that compared with cells without treatment, NOX4 and p–p38 expression levels were increased in cells transduced with oe-NC in the presence of DMSO, while p–p38 expression levels were reduced following SB203580 treatment, whereas NOX4 and p–p38 expression levels were increased after treatment with oe-NOX4 and DMSO. In addition, compared with oe-NOX4 and DMSO, p–p38 expression levels were decreased by oe-NOX4 in combination with SB203580 treatment (Fig. 7B). Meanwhile, compared with untreated cells, it was found that cell viability was decreased and apoptosis was increased following oe-NC and DMSO treatment, whereas cell viability was promoted and apoptosis was repressed as a result of oe-NOX4 and DMSO treatment, and these trends were reversed when further transduced with SB203580 (Fig. 7C, D). The results of SOD detection, MDA detection, and DCFH-DA fluorescent staining illustrated that compared with cells without treatment, SOD levels were decreased and the levels of MDA and ROS were increased following oe-NC and DMSO treatment. Compared with oe-NC + DMSO treatment, SOD levels were increased and MDA and ROS levels were decreased by oe-NOX4 in the presence of DMSO, while these trends were reversed after further transduction with SB203580 (Fig. 7E–G).

### MiR-181a–2–3p inhibits OS injury of SH-SY5Y cells via EGR1/NOX4 axis

**Fig. 8:**
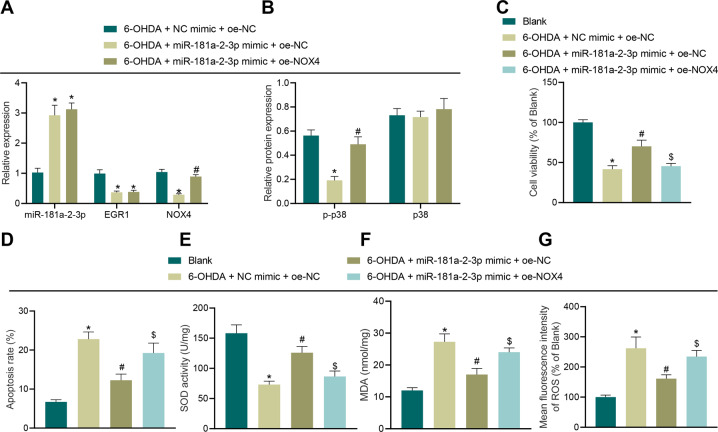
miR-181a–2–3p inhibits apoptosis and OS of SH-SY5Y cells by regulating the EGR1/NOX4 axis. **A** miR-181a–2–3p, EGR1, and NOX4 expression in SH-SY5Y cells treated with 6-OHDA in combination with different plasmids determined using RT-qPCR. **B** Levels of p–p38 and p38 in SH-SY5Y cells treated with 6-OHDA in combination with different plasmids determined using Western blot analysis. **C** Cell proliferation in SH-SY5Y cells treated with 6-OHDA in combination with different plasmids. **D** Cell apoptosis in SH-SY5Y cells treated with 6-OHDA in combination with different plasmids. **E** SOD level in SH-SY5Y cells treated with 6-OHDA in combination with different plasmids detected using SOD kit. **F** MDA level in SH-SY5Y cells treated with 6-OHDA in combination with different plasmids detected using MDA kit. **G** ROS levels in SH-SY5Y cells treated with 6-OHDA in combination with different plasmids detected using DCFH-DA fluorescent staining. **C**, **E**, **F**, and **G**, *p < 0.05 vs. NC mimic + oe-NC or SH-SY5Y cells without treatment, ^#^*p* < 0.05 vs. miR-181a–2–3p mimic + oe-NC or NC mimic + oe-NC; ^$^*p* < 0.05 vs. miR-181a–2–3p mimic + oe-NC. Cell experiments were repeated three times independently.

As demonstrated by the results of RT-qPCR and Western blot analysis, miR-181a–2–3p expression levels were increased, while those of EGR1, NOX4 and p–p38 were all decreased in cells transduced with miR-181a–2–3p mimic alone, while additional treatment of oe-NOX4 elevated the expressions of NOX4 and p–p38 (Fig. 8A, B). In addition, compared with untreated cells, it was found that cell viability was decreased and apoptosis was increased after transduction with NC mimic and oe-NC in combination. Meanwhile, cell viability was increased and apoptosis was reduced following transduction with miR-181a–2–3p mimic alone, while these trends were abrogated after treatment with oe-NOX4 (Fig. 8C, D). The results of SOD detection, MDA detection, and DCFH-DA fluorescent staining illustrated that compared with cells without treatment, SOD levels were decreased and the levels of MDA and ROS were increased by NC mimic and oe-NC treatment. On the other hand, SOD levels were elevated and MDA and ROS levels were reduced by miR-181a–2–3p mimic alone, while these trends were reversed after transduction with oe-NOX4 (Fig. 8E–G).

### MSC–EVs carrying miR-181a–2–3p relieve symptoms of PD in mice

**Fig. 9:**
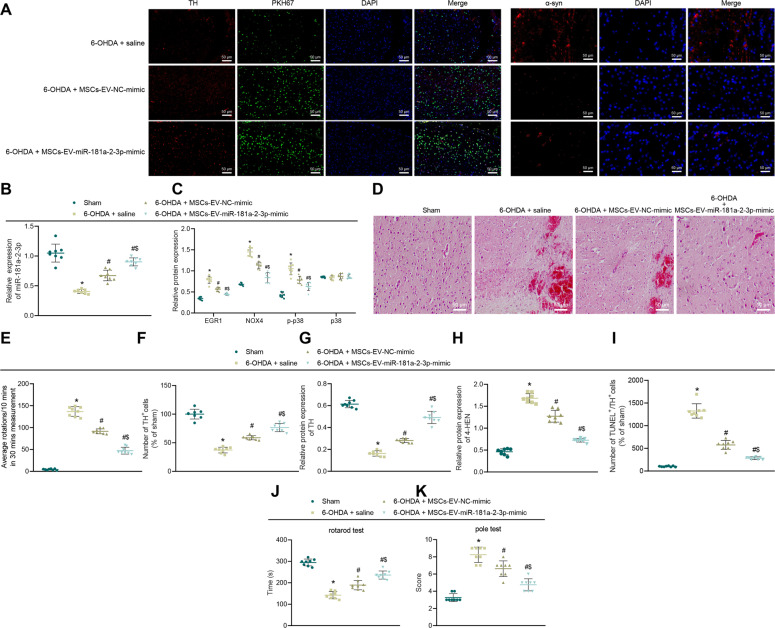
MSC–EVs carrying miR-181a–2–3p inhibit neuronal loss and OS injury in the SN area of PD mice. **A** The PKH67-labeled EVs were injected intravenously into mice. Colocalization of EVs (green) with dopaminergic neurons in the SN tissues (the left panel). *α*-SYN expression (red) determined by immunofluorescence. DAPI (blue) located in the nucleus (scale bar = 15 μm). **B** miR-181a–2–3p expression in SN tissues detected using RT-qPCR. **C** EGR1, NOX4, p–p38, and p38 levels in SN tissues detected using Western blot analysis. **D** Pathological changes of SN tissues determined with H&E staining. The arrow represents the lost neurons (200 ×). **E** APO-induced asymmetric rotation in PD mice injected with EVs after 8 weeks. **F** TH expression in mouse SN tissues detected by immunohistochemical staining. **G** TH expression in mouse SN tissues detected using Western blot analysis. **H** OS marker 4-HNE in mouse SN tissues. **I** The dopaminergic neuron apoptosis in SN tissues detected by immunofluorescence staining. **J** The motor ability of mice detected by roller test. **K** The motor ability of mice determined by rod-climbing pole test *p < 0.05 vs. sham-operated mice, ^#^*p* < 0.05 vs. saline; ^$^*p* < 0.05 vs. MSC–EV–NC mimic.

To investigate whether MSC–EVs carrying miR-181a–2–3p can alleviate PD in vivo, we further established PD-modeled mice. To determine whether MSC–EVs can reach the substantia nigra (SN) through the blood–brain barrier, we injected normal saline and PKH67-labeled MSC–EVs into PD mice. In the 6-OHDA-induced PD mice injected with saline, the intensity of green fluorescence and colocalization of SN dopaminergic neurons were found to be stronger, while the expression levels of alpha-synuclein (α-syn) (pathological fibers in PD) were reduced in 6-OHDA-treated mice injected with MSC–EV–NC mimic or MSC–EV–miR-181a–2–3p mimic (Fig. 9A). In mouse brain tissue SN, miR-181a–2–3p expression levels were decreased, while those of EGR1, NOX4, and p–p38 were all increased in PD mice injected with saline relative to sham-operated mice. Compared with saline treatment, miR-181a–2–3p expression levels were increased and those of EGR1, NOX4, and p–p38 were decreased in PD mice injected with MSC-EV-NC-mimic or MSC-EV-miR-181a–2–3p mimic. Meanwhile, compared with MSC–EV–NC mimic, miR-181a–2–3p expression levels were elevated and those of EGR1, NOX4, and p–p38 were all reduced in PD mice injected with MSC–EV–miR-181a–2–3p mimic (Fig. 9B, C; Supplementary Fig. 3C).

Furthermore, the results of hematoxylin–eosin (HE) staining of SN sections illustrated the presence of scattered large multipolar neurons in the dense cells of sham-operated mice, with a vesicle-like nucleus and surrounding nerve-fiber network; the cells in PD mice injected with saline were significantly twisted, angulated, and atrophied, in addition to vacuoles in the intercellular space, as well as different signs of degeneration and necrosis, manifested as deep eosinophilic cytoplasm, accompanied by nuclear pyknosis and nuclear lysis changes; the symptoms of PD mice injected with MSC–EV–NC mimic were found to be alleviated, with multipolar neurons with nucleolus and basophilic granular cytoplasm in the dense part of SN; mice injected with MSC–EV–miR-181a–2–3p mimic exhibited a large number of multipolar neurons with nucleolus and basophilic granular cytoplasm (Fig. 9D).

Additionally, we evaluated the asymmetric rotation induced by apomorphine (APO) at the 8th week after EV treatment. It was found that compared with sham-operated mice, contralateral rotation was increased in PD mice; compared with saline, contralateral rotation of animals was reduced in PD mice injected with MSC–EV–NC mimic or MSC–EV–miR-181a–2–3p mimic. Meanwhile, compared with MSC–EV–NC mimic, contralateral rotation of animals was observed to be reduced in PD mice injected with MSC–EV–miR-181a–2–3p mimic (Fig. 9E). Moreover, immunohistochemical detection of the dopaminergic neuron marker TH in mouse SN tissue depicted that compared with sham-operated mice, the dopaminergic neurons were severely damaged in PD mice. Meanwhile, relative to saline treatment, damage to dopaminergic neurons was alleviated in PD mice injected with MSC–EV–NC-mimic or MSC–EV–miR-181a–2–3p mimic. Compared with MSC–EV–NC mimic, dopaminergic neurons were increased by MSC–EV–miR-181a–2–3p mimic (Fig. 9F; Supplementary Fig. 3D).

Moreover, we found that TH expression levels were reduced in PD mice relative to sham-operated mice. Compared with saline treatment, TH expression levels were increased in PD mice injected with MSC–EV–NC mimic or MSC–EV–miR-181a–2–3p mimic. Meanwhile, when compared with MSC–EV–NC mimic, TH expression levels were increased in PD mice injected with MSC–EV–miR-181a–2–3p mimic (Fig. 9G; Supplementary Fig. 3E). The results of Western blot analysis further demonstrated that 4-HNE expression levels were increased in PD mice relative to sham-operated mice. Compared with saline, 4-HNE expression levels were decreased in PD mice injected with MSC–EV–NC mimic or MSC–EV–miR-181a–2–3p mimic. In addition, when compared with MSC–EV–NC mimic, 4-HNE expression levels were found to be reduced in PD mice injected with MSC–EV–miR-181a–2–3p mimic (Fig. 9H; Supplementary Fig. 3F). TUNEL and TH double labeling were employed to validate the effect of EVs on apoptosis of dopaminergic neurons, the results of which demonstrated that the number of TUNEL^+^ cells in sham-operated mice was very small, while the number of TUNEL^+^/TH^+^ cells was reduced in PD mice injected with MSC–EV–NC mimic, and the number being obviously decreased in PD mice injected with MSC–EV–miR-181a–2–3p mimic (Fig. 9I).

As demonstrated by the results of rod-climbing test and roller test, compared with sham-operated mice, the falling time was reduced, while the time required for pole-climbing test from top to bottom was increased in the PD mice, whereas further injection with MSC–EV–NC mimic or MSC–EV–miR-181a–2–3p mimic reversed these trends. Meanwhile, compared with MSC–EV–NC mimic, the falling time was increased, while the time required for pole-climbing test from top to bottom was declined in PD mice injected with MSC–EV–miR-181a–2–3p mimic (Fig. 9J, K). Collectively, these findings indicated that MSC–EVs carrying miR-181a–2–3p could relieve the symptoms of PD in mice.

## Discussion

Returning to our initial hypothesis that transfer of miR-181a-5p via MSC-derived EVs might alter OS in PD, we came across evidence in our study revealing that miR-181a–2–3p shuttled by MSC-derived EVs could be transferred into SH-SY5Y cells, where miR-181a–2–3p exerted inhibitory effects on OS in NAFLD via downregulation of the EGR1/NOX4/p38 MAPK axis.

Initial findings in our study demonstrated that miR-181a–2–3p was poorly expressed in induced PD cells, which is line with a prior PD-focused study [13]. In addition, we observed that miR-181a–2–3p from MSCs could be transferred to SH-SY5Y cells via EVs, in order to augment SH-SY5Y proliferation and SOD levels, while suppressing apoptosis and the levels of MDA and ROS, so as to relieve OS in PD in vitro. OS is particularly important in regard to PD, as the imbalance can induce apoptosis and impair the autophagy of dopaminergic neurons in PD [11]. Moreover, prior studies have shown that the amelioration of OS is accompanied by promoted viability, decreased apoptosis of neurons, decreased MDA and ROS, and increased SOD levels [21]. Meanwhile, pretreatment with EVs is known to enhance PD cell proliferation and reduce substantia nigra dopaminergic neuronal loss and apoptosis, by virtue of inducing autophagy to relieve PD [7]. Inherently, EVs possess the ability to transport a number of small biological molecules, including miRNAs, to surrounding cells [22]. Elaborating on the same, dysregulation of miRNAs can result in mitochondrial dysfunction, OS, and cell death, subsequently causing neurodegeneration in PD [23]. One such miRNA, namely miR-181a, was previously identified as a PD biomarker, such that miR-181a was poorly expressed in PD, while aberrant miR-181a expressions in PD were indicated to regulate neuronal growth to affect PD progression [24]. Furthermore, miR-181c has been shown to promote cell viability and inhibit apoptosis to suppress the development of PD, which is in accordance with our findings [25]. Additionally, a prior study illustrated that upregulation of miR-375 leads to the attenuation of neuroinflammatory response and OS and inhibition of apoptosis, thus serving as a peripheral diagnostic biomarker and therapeutic target for PD [26]. Collectively, these findings and evidence supported that miR-181a–2–3p shuttled by EVs from MSCs was poorly expressed, while restoration of miR-181a–2–3p could induce cell proliferation and inhibit apoptosis and OS, thereby suppressing the progression of PD. Moreover, in vivo findings in our study revealed that EV containing miR-181a–2–3p overexpression reduced the expression of α-syn and decreased 4-HNE in PD mice, which is particularly important as 4-HNE is known to serve as a marker of OS [27]. Meanwhile, accumulation of *α*-syn fibrils in neuronal inclusions is regarded as a hallmark for the pathology of PD [28].

Additionally, our findings further demonstrated that miR-181a–2–3p could target EGR1 to inhibit its expression. In line with our discovery, another study previously documented that miR-181a-5p overexpression could directly suppress EGR1 [14]. Moreover, EGR1 further plays a role in PD, as demonstrated by their ability to induce neuronal death, neuroinflammation and dopaminergic cell-body loss [16]. Furthermore, our findings revealed that EGR1 is enriched in the promoter region of NOX4, and consequently promotes NOX4, which is relevant as another research came across upregulated levels of NOX4 in the brains of PD patients [18]. High expression of NOX4 is further known to contribute to OS to induce cell damage [19, 29]. It is also noteworthy that upregulation of NOX4 can induce Aβ expression to suppress cognitive function in the hippocampal DG of 6-OHDA-treated mice [20]. In addition, we learnt that NOX4 activated the p38 MAPK pathway to aggravate OS in PD, whereas previous studies have shown that restoration of NOX4 is conducive to the activation of p38 MAPK pathway [30]. Besides, activation of MAPK signaling like p38 is well-documented in PD [31]. Meanwhile, p38 MAPK is further implication in various processes in PD, such as neuronal inflammation, apoptosis, and OS [32]. p38 MAPK is able to enhance neuronal apoptosis in PD [33], and its activation could facilitate OS in PD [34]. Liu et al. have also confirmed that miR-181a inhibits apoptosis and autophagy in PD by inhibiting the p38 MAPK pathway, which is much in line with our findings [13]. In line of these findings, it would be plausible to suggest that miR-181a–2–3p shuttled by MSC-secreted EVs inhibits OS in PD by inhibiting the EGR1/NOX4/p38 MAPK axis.

**Fig. 10:**
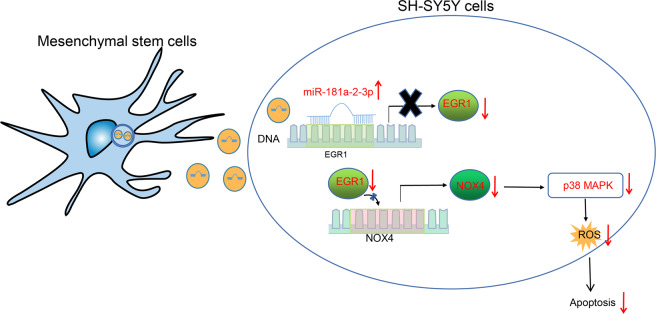
MSC–EVs carrying miR-181a–2–3p into SH-SY5Y cells inhibit EGR1 to suppress NOX4 transcription and activation of p38 MAPK pathway, thereby repressing the apoptosis and OS of SH-SY5Y cells in PD.

To sum up, herein, we uncovered that transfer of miR-181a–2–3p via MSC-derived EVs altered the NOX4/p38 MAPK expression by negatively regulating EGR1, all of which leads to the inhibition of OS in PD (Fig. 10). Our findings pave the way for effective therapeutic strategies for inhibiting OS in PD. However, due to the lack of present researches, the roles of miR-181a–2–3p shuttled by MSC-derived EVs, EGR1, as well as their interaction in the OS in the progression of PD require further exploration. Moreover, in vivo imaging detection of mice should be performed in the future to clinically validate our findings.

## Materials and methods

### Bioinformatic analysis

First, miRNAs related to PD were identified with the help of existing literature, and subsequently, the PD-associated miRNA expression dataset GSE16658 deposited in the GEO database was extracted and analyzed using the R language. There were 32 samples in the GSE16658 dataset, comprising 13 normal samples and 19 PD samples. The enrichment of miRNA in various EVs was detected by the EVmiRNA database. Furthermore, Diana TOOLS (miTG score >0.35) and microRNA (energy < −15, mirsvr_score < −0.6) were adopted to predict the miRNA downstream genes, which were intersected with the human transcription factors obtained from hTFtarget, Cistrome, and JASPAR databases to determine the downstream transcription factors of miRNA. A protein-interaction network was then constructed with String (minimum required interaction score: 0.4), and the core degree was calculated using Cytoscape to determine the most critical transcription factors. In addition, the binding sites of key transcription factors targeted by miRNA were obtained from DIANA TOOLS. Moreover, the downstream pathway of transcription factors was determined by Coexpression analysis from MEM and existing literature.

### Cell culture and transfection

Human neuroblastoma cell line SH-SY5Y was procured from the Cell Bank of Chinese Academy of Sciences (Shanghai, China), and cultured in DMEM (30030, Gibco, Carlsbad, California, USA) supplemented with 100 U/mL penicillin and 100 U/mL streptomycin (15070063, Gibco) and 10% fetal bovine serum (FBS, 16140089, Gibco). The cells were cultured in a humid incubator containing 5% CO_2_ at 37 °C.

SH-SY5Y cells (4 × 10^5^ cells/well) were subsequently seeded in a 6-well plate, and transduced using Lipofectamine 2000, and plasmids were purchased from Shanghai GenePharma Co. Ltd. (Shanghai, China). After culturing for 48 h, 6-OHDA was added at the specified concentration for 24 h. Cells in the designated groups were treated with p38 inhibitor SB203580 (10 μmol/L, Sigma-Aldrich, St. Louis, USA) [31]. In the subsequent groups, a dosage of 100 μM was selected for the 6-OHDA treatment, and the cells were divided into the following groups: blank (no treatment), 6-OHDA (6-OHDA treatment), 6-OHDA + NC mimic (6-OHDA treatment + transduced with NC mimic), 6-OHDA + miR-181a–2–3p mimic (6-OHDA treatment and transduced with miR-181a–2–3p mimic), NC mimic (transduced with NC mimic), miR-181a–2–3p mimic (transduced with miR-181a–2–3p mimic), NC inhibitor (transduced with NC inhibitor), miR-181a–2–3p-inhibitor (transduced with miR-181a–2–3p inhibitor), 6-OHDA + NC inhibitor + si-NC (6-OHDA treatment + transduced with NC inhibitor and si-NC), 6-OHDA + NC inhibitor + si-EGR1 (6-OHDA treatment + transduced with NC inhibitor and si-EGR1), 6-OHDA + miR-181a–2–3p inhibitor + si-EGR1 (6-OHDA treatment + transduced with miR-181a-2-3p inhibitor and si-EGR1), 6-OHDA + oe-NC + si-NC (6-OHDA treatment)+ transduced with oe-NC + si-NC, 6-OHDA + oe-NC + si-NOX4 (6-OHDA treatment + transduced with oe-NC + si-NOX4), 6-OHDA + oe-EGR1 + si-NOX4 (6-OHDA treatment + transduced with oe-EGR1 + si-NOX4), 6-OHDA + oe-NC + DMSO (6-OHDA + DMSO treatment + transduced with oe-NC), 6-OHDA + oe-NC + SB203580 (6-OHDA + SB203580 treatment + transduced with oe-NC), 6-OHDA + oe-NOX4 + DMSO (6-OHDA + DMSO treatment + transduced with oe-NOX4), 6-OHDA + oe-NOX4 + SB203580 (6-OHDA + SB203580 treatment + transduced with oe-NOX4), 6-OHDA + NC mimic + oe-NC (6-OHDA treatment + transduced with NC mimic + oe-NC), 6-OHDA + miR-181a–2–3p mimic + oe-NC (6-OHDA treatment + transduced with miR-181a–2–3p mimic + oe-NC), and 6-OHDA + miR-181a–2–3p mimic + oe-NOX4 (6-OHDA treatment + transduced with miR-181a–2–3p mimic + oe-NOX4) groups.

### Isolation and culture of mouse primary neuron cells

The whole body of mice was wiped with alcohol, the head was cut off, and the scalp and skull were carefully peeled off to collect the intact brain tissues, which were placed in ice-cold PBS. Subsequently, the meninges were carefully peeled off using pointed tweezers, and the blood vessels in the cerebellum and brain were collected in EP tubes. The tissues were repeatedly cut and incubated with prewarmed trypsin for 20 min. After the digestion was completed, serum-containing DMEM was added to terminate digestion, and pipetting was repeated several times. The tissue mass was removed using a 200-mesh sieve. The following day, the medium was renewed to neuron culture medium and added with 5-fluoro-deoxyuridine after 48 h.

### Extraction and identification of MSC–EVs

Healthy human umbilical cord mesenchymal stem cells (hucMSCs) (Yiyan Biotechnology, Shanghai, China) were cultured overnight in DMEM without EVs. Upon reaching 80–90% cell confluency, the supernatant was centrifuged and precipitated in a serum-free DMEM containing 25 mM HEPES (pH = 7.4), and centrifuged again. The obtained precipitate was stored at −80 °C.

TEM was adopted to identify EVs. Briefly, 10 μL of EVs were allowed to stand for 1 min. Next, the EVs were counter-stained with 30 μL of PTA (pH = 6.8) for 5 min, and an incandescent lamp was used to bake-dry the EVs, and photographs were taken under the TEM.

In short, the Zetasizer Nano-ZS90 instrument (Malvern Instruments, Worcestershire, UK) was adopted. The EV sample was diluted with 0.15 M NaCl (1:50) for detection.

The EVs were dissolved in RIPA and quantitatively identified. The antibodies used were purchased from Abcam (Cambridge, UK): CD9 (ab92726, dilution ratio of 1:1000), CD63 (ab134045, dilution ratio of 1:1000), TSG101 (ab125011, dilution ratio of 1:2000), and calinexin (ab22595, dilution ratio of 1:1000).

### EV-uptake assay

Purified EVs were mixed and incubated with PHK67 (MINI67, Sigma-Aldrich) for 5 min. The staining was stopped with the addition of 2 mL of 10% bovine serum albumin (BSA). Subsequently, EV-free serum medium was supplemented to 8.5 mL, and 1.5 mL of 0.971 M sucrose was slowly added from the bottom of the test tube to ensure that PKH-67-EVs were on top of the sucrose solution. Following centrifugation, EV pellets were resuspended in PBS. Next, SH-SY5Y cells were cultured, the medium was renewed for 48 h, and added with PKH67-labeled EVs at 37 °C for 6 h. Later, the cells were fixed with 4% polytetrafluoroethylene for 15 min, stained with 0.1 g/mL DAPI (C1002, Beyotime, Nantong, China) for 5 min, and then SH-SY5Y cells were placed in a fluorescence microscope (Olympus, Tokyo, Japan) to detect the fluorescence expression.

### Cy3-labeled miR-181a–2–3p transfection

The pre-miR miRNA precursor (miR-181a–2–3p, Ambion, Austin, Texas, USA) was labeled with Label IT provided by siRNA Tracker Cy3 kits (Mirus, Madison, Wisconsin). The MSCs (1 × 10^5^) were transfected with 10 nM Cy3-labeled pre-miR miRNA precursor. After that, the medium was renewed with fresh serum-free AIM V medium (Invitrogen) and used to prepare EVs one day after cultivation.

### Absolute quantitative detection of miRNA

The concentration of miRNA, measured with the help of nucleic acid A260 nm spectrophotometry, was converted into copy number to prepare a standard curve. Following RT-qPCR, its PCR signal (crossover point, Cp) was interpolated into the standard curve using the LightCycler^®^480 software 1.5 (Roche, Basel, Switzerland) to determine the concentration of miRNA. The miRNA presenting with the highest degree of enrichment was selected, and the copy number of each explant was quantified using the RT-qPCR absolute-quantification method. In addition, synthetic nonhuman Xaer-miR-39 was added during RNA-isolation process. The copy of cel-miR-39 was quantified using absolute quantification of RT-qPCR to evaluate the similar recovery efficiency of cel-miR-39 in the test samples. As a result, there was no deviation in the quantification of miR-181a–2–3p. Afterward, the number of explant particles in each formulation was measured by means of nanoparticle-tracking analysis (NTA) or qNano. We chose two independent quantifications to overcome the lack of optimal quantification criteria for explant particles.

### Radioactive dopamine-uptake test

Cells seeded in a 24-well plate were incubated with 16 nM [3H]dopamine (32,6 Ci/mmol Perkin Elmer, Waltham, MA). The uptake reaction was then stopped. In order to release the ingested [3H]dopamine, the cells were incubated with 500 μL of 96% (v/v) ethanol at 37 °C for 30 min. Subsequently, an aliquot of 5 mL scintillation fluid was added to the vial to evaluate the radioactivity in a scintillation counter.

### MTT assay

Cells (8 × 10^4^ cells/well) seeded in a 24-well plate were incubated with 0.5 mg/mL MTT reagent for 2 h at humid atmosphere containing 5% CO_2_ at 37 °C. After the medium was removed, the formazan crystals were dissolved with 200 μL of solubilization buffer [20% SDS (pH 4.7)] within necessary time. Afterward, the chromaticity was measured with a spectrophotometer at a wavelength of 570 nm.

### Detection of intracellular oxidation level

The H2DCFDA probe was adopted to assess the cellular oxidant content, which could pass through the membrane, and the probe could be converted into a fluorescent compound after oxidation. Following treatment, the culture medium was renewed with DMEM medium containing 10 μM H2DCFDA. After incubation, the cells were lysed in a buffer containing PBS and 1% NP-40. Afterward, the fluorescence in the lysate (*λ*_ex_ = 495, *λ*_em_ = 530) was measured using a Fluoroskan Ascent FL microplate fluorometer.

### Lactate-dehydrogenase (LDH) release assay

After cell treatment, the medium was centrifuged at 1000 × *g* for 10 min. Subsequently, LDH activity in the obtained supernatant was determined using spectrophotometry with LDH-P UV AA kits, according to the manufacturer’s instructions. In short, the conversion of reduced nicotinamide adenine dinucleotide to oxidized nicotinamide adenine dinucleotide was tracked at a wavelength of 340 nm. The results were expressed as a percentage of the control.

### Isolation and quantification of RNA

Total RNA content was extracted from SH-SY5Y cells or EVs using the TRIzol reagent (Catalog 16096020, Thermo Fisher, Austin, Texas, USA). Reverse-transcription kits (RR047A, Takara, Otsu, Shiga, Japan) were adopted for mRNA detection. Poly-A tailing detection kits (B532451, Sangon, Shanghai, China; containing universal PCR primer R and U6 universal PCR primer R) was further used for miRNA. SYBR^®^ Premix Ex Taq^TM^ II kit (DRR081, Takara) were used for sample loading, and RT-qPCR was performed in fluorescent quantitative PCR instrument. U6 mRNA levels were used as the internal control. For miRNA in EVs, syn-cel-miR-39 was employed as an endogenous control for data normalization. The primer sequences are listed in Supplementary Table 1. The 2^−ΔΔCt^ method was used.

### Western blot

Total protein content was extracted from SH-SY5Y cells or EVs using RIPA kits (R0010, Solarbio, Beijing, China). Briefly, 40 µg of each sample was extracted, separated with 10% SDS-PAGE, and electrotransferred onto a PVDF membrane (Merck Millipore, Billerica, MA, USA). Subsequently, the membrane was blocked and incubated with the following primary antibodies: EGR1 (dilution ratio of 1:1000, #4154, CST, Beverly, MA, USA), NOX4 (dilution ratio of 1:2000, ab109225, Abcam), p–p38 (dilution ratio of 1:1000, #4511, CST), P38 (dilution ratio of 1:1000, #8690, CST), TH (dilution ratio of 1:5000, ab137869, Abcam), 4-HNE (dilution ratio of 1:1000, ab46545, Abcam), and GAPDH (dilution ratio of 1:10000, ab128915, Abcam). Afterward, the membrane was incubated with IgG (dilution ratio of 1:10000, ab97051, Abcam). Later, the membrane was developed using an electrogenerated ECL, and then the membrane was exposed to light using the Image Quant LAS 4000 C gel imager (GE Company, Schenectady, NY, USA) and analyzed with the ImageJ software (1.48 u, National Institutes of Health, Maryland).

### Chromatin-immunoprecipitation (ChIP) assay

ChIP assay was carried out with the help of ChIP kits (Merck Millipore). Briefly, SH-SY5Y cells were taken from each group, and added with 1% formaldehyde upon reaching 70–80% cell confluency. Next, the cells were fixed to make intracellular DNA and protein cross-linking, and then the fixing was stopped with the addition of glycine. Subsequently, the complex was lysed at 4 °C and randomly fragmented into 500–1000 bp fragments by means of ultrasonification. The obtained supernatant was collected and divided into three tubes. The positive-control antibody RNA polymerase II, negative-control antibody normal rabbit IgG, and rabbit anti-EGR1 (dilution ratio of 1: 50, #4154, CST) were added. Protein Agarose/Sepharose was used to precipitate endogenous DNA–protein complexes, which was de-cross-linked at 65 °C. DNA fragments were purified by phenol/chloroform. NOX4 promoter expression was tested using RT-qPCR and primers are shown in Supplementary Table 2.

### Dual-luciferase reporter gene assay

A dual-luciferase reporter gene assay was adopted to verify whether EGR1 could be targeted by miR-181a–2–3p, and human EGR1 3′UTR WT fragments containing miR-181a–2–3p binding sites were synthesized. Endonuclease sites, *SpeI* and *Hind III*, which introduced pMIR reporter, were obtained from Huayueyang Biotechnology, Beijing, China. MUT sites were designed based on the EGR1 WT 3′UTR and miR-125a-5p binding site, and the target fragment was subsequently inserted into the pMIR-reporter plasmid. Lipofectamine 3000 (Invitrogen) was employed to cotransfect the plasmids with miR-181a–2–3p mimic or NC-mimic into HEK293T cells. The luciferase activity at a wavelength of 570 nm was detected using a dual-luciferase reporter system (Promega, Madison, WI) and Glomax20/20 luminometer fluorescence detector (Promega). Relative luciferase activity = Firefly luciferase activity/Renilla luciferase activity

### CCK-8 assay

SH-SY5Y cells (at 1 × 10^3^ cells/well) were seeded in a 96-well plate and cultured for 1–5 days, and cell-proliferation activity of SH-SY5Y in different groups was subsequently tested according to the manufacturer’s instructions of the CCK-8 kit (K1018, Apexbio, Boston, MA, USA). Afterward, 10 μL of CCK-8 solution was added and incubated for 1 h, and absorbance was measured at a wavelength of 450 nm.

### Flow cytometry

AnnexinV/PI apoptosis-detection kits (BD Biosciences, Franklin Lakes, NJ) were adopted to detect apoptosis rate. Briefly, the cells were resuspended in binding buffer and incubated with 5 μL of fluorescein isothiocyante (FITC)–Annexin V and 5 μL of PI for 15 min, and the FACScan flow-cell flow system (Becton Dickinson, San Diego, CA, USA) was used for apoptosis detection.

### ROS detection

Different kits provided by Jiancheng Bioengineering Institute (Nanjing, China) were employed for the detection of SOD levels (Cat. No.: A001-3) and MDA levels (Cat. No.: A003-1) in cell culture medium. Cells in each group were seeded in 24-well plates, incubated with 200 μL of serum-free DCFH-DA, and fluorescence was subsequently detected by means of flow cytometry.

### Construction of PD mouse models

Injections of 6-OHDA into the right striatum were used to establish a PD mouse model. Briefly, the mice were placed in a stereotaxic device (Neurostar, Tubingen, Germany) and anesthetized with 2% isoflurane. The coordinates of the same insertion point and different depths in the right striatum were as follows: AP–0.2 mm, ML–3 mm, DV–4.5 mm, AP–0.2 mm, ML–3 mm, and DV–5.5 mm. Subsequently, the mice were stereotactically injected with 6-OHDA (3 μL of 5 μg/μL 6-OHDA solution containing 0.2% ascorbic acid). Meanwhile, the sham operated mice were injected with 0.2% ascorbic acid normal saline (*n* = 8). APO-induced rotation is regarded as an ideal predictor of maximum dopamine consumption in the unilateral striatum to evaluate and screen 6-OHDA-induced PD mice [35]. On the 4^th^ week post operation, the mice were injected with 0.5 mg/kg APO intraperitoneally. After 5 min of APO administration, rotation data were recorded continuously for 30 min. The mice with 7 laps per min were regarded as successfully modeled mice [36]. The successfully modeled mice were then randomly divided into three groups (*n* = 8), and treatment was initiated at the 4^th^ week after the operation as follows: (1) in the 6-OHDA + saline group, each mouse was administered 0.5 mL of normal saline through the tail vein, which exceeded 5 min and the flow rate did not exceed 0.1 mL/min; (2) the 6-OHDA + MSC–EV–NC mimic (6-OHDA-induced PD mice were injected with MSC–EV via tail vein, with a concentration of 200 g/0.5 mL saline, and a flow rate not exceeding 0.1 mL/min) group; (3) the 6-OHDA + MSC–EV–miR-181a–2–3p mimic group (6-OHDA-induced PD mice were injected with MSC–EV–miR-181a–2–3p mimic with a concentration of 200 µg/0.5 mL saline and a flow rate not exceeding 0.1 mL/min). The mice were treated as above-mentioned once every 3 days for a duration of 8 weeks. The Animal Ethics Committee of Henan Provincial People’s Hospital, (Zhengzhou University People’s Hospital) (2019-067 A) also provided ethical approval for animal experimentation in the current study.

### HE staining

Routinely treated brain tissues from mice were sliced into 3 small sections, deparaffinized, rehydrated, and then stained with Mayer’s hematoxylin. Next, the sections were simply distinguished four times with acidic alcohol, blued, and observed under a microscope. Following observation, the sections were dehydrated, stained with eosin, and sealed.

### In vivo imaging detection

Initially, 25 mg of Xenolight DiR fluorescent dye (PerkinElmer, Waltham, MA) was dissolved in 2 mL of ethanol. Next, the separated 0.5 mL MSC–EVs were diluted with 0.5 mL of PBS and incubated with DiR stock solution for 30 min. The sample was centrifuged a 100,000 × *g* for 60 min, the supernatant was discarded, and 300 μL of PBS was adopted to dissolve the blue precipitate in the lower layer. Subsequently, the DiR-labeled EVs were injected into mice via tail-vein injection. After 24 h, the analysis was performed using the caliper IVIS Lumina II imager.

### Immunofluorescence

Paraffin-embedded brain tissues were sliced into 6 μm sections, and then subjected to immunofluorescence staining. After 5 min of antigen retrieval, the sections were incubated in 5% goat serum for 1 h and incubated with rabbit TH (dilution ratio of 1:500, GB11181, Servicebio, Wuhan, China) and 4-HNE (dilution ratio of 1:50, MA5-27570, Invitrogen), and α-SYN (dilution ratio of 1:500, ab272736, Abcam) at 4 °C overnight and with Cy3-conjugated donkey anti-rabbit antibody (dilution ratio of 1:250, GB 21403, Servicebio) or Goat Anti-Mouse Alexa Fluor 488 (dilution ratio of 1:1000, A32723, Invitrogen) for 1 h. A confocal laser microscope (Olympus) was employed to observe the immune-response cells.

### Immunohistochemistry

Immunohistochemistry was routinely performed [7]. Primary antibody TH (dilution ratio of 1:500, ab137869, Abcam) was added dropwise in the refrigerator at 4 °C overnight.

### TUNEL assay

SN cell apoptosis was quantitatively detected using In Situ Cell Death Detection kits (Roche Diagnostics GmbH, Mannheim, Germany). In short, dewaxing procedure was performed as mentioned above. Next, the membrane was broken using a membrane-breaking solution. Subsequently, TUNEL and TH (dilution ratio of 1:500, Servicebio) were used to determine the percentage of apoptotic dopaminergic neurons. Dopaminergic neurons were then incubated with the secondary antibody for 50 min. Following staining with DAPI, fluorescein-labeled green DNA and red dopaminergic neurons are observed under a fluorescence microscope.

### Behavioral experiment

Behavior changes of mice were observed with the help of rod-climbing test and roller test. First, the rod-climbing test was performed. Briefly, a 50-cm-long and 1-cm-diameter wooden pole wese placed vertically, wrapped with gauze to prevent mice slipping off, and a 2.5-cm-diameter cork ball were fixed on the top. The mice were placed on the cork ball, and the time of climbing from the top of the ball to the top of the rod, from the top of the rod to the middle of the rod, and from the middle of the rod to the bottom of the rod were all recorded. Completion within 3 sec was recorded as 3 points, and 6 sec as 2 points. If the time exceeded 6 sec or the mouse fell from the pole, the attempt would be recorded as 1 point. Experiment results were expressed by the total score. Meanwhile, for the roller test, the mice were placed in a roller experimental device at 4 r/min for 2 min for adaption. The axis experimental device mode was set as acceleration 4 ~ 40 r/min, and the time from the acceleration of the roller to the mouse falling off the device was recorded, with the average value of three times obtained. The time interval of each measurement is 1 h.

### Statistical analysis

Data analyses were performed using the GrafPad Prism software (GraphPad Software, La Jolla, CA). All quantitative data were presented as mean ± standard deviation. Comparisons between two groups were analyzed with an independent-sample *t-*test. One-way analysis of variance (ANOVA) with post hoc Tukey test was used for comparisons between multiple independent groups. The dual-luciferase reporter gene assay was analyzed using two-way ANOVA with Bonferroni as the post hoc. A value of *p* < 0.05 was regarded statistically significant.

## Supplementary information

Supplementary Figures

Supplementary Tables

## Data Availability

The datasets generated/analyzed during the current study are available.
