# Supplementary material for: MicroRNA-181a–2–3p shuttled by mesenchymal stem cell-secreted extracellular vesicles inhibits oxidative stress in Parkinson’s disease by inhibiting EGR1 and NOX4
Source: Cell Death Discov. 2022 Jan 24;8:33. doi: 10.1038/s41420-022-00823-x (PMC8786891; doi:10.1038/s41420-022-00823-x)

**Supplementary Fig. 1.** The enrichment of miR-181a-2-3p in EVs sourced from different cells predicted in the database.


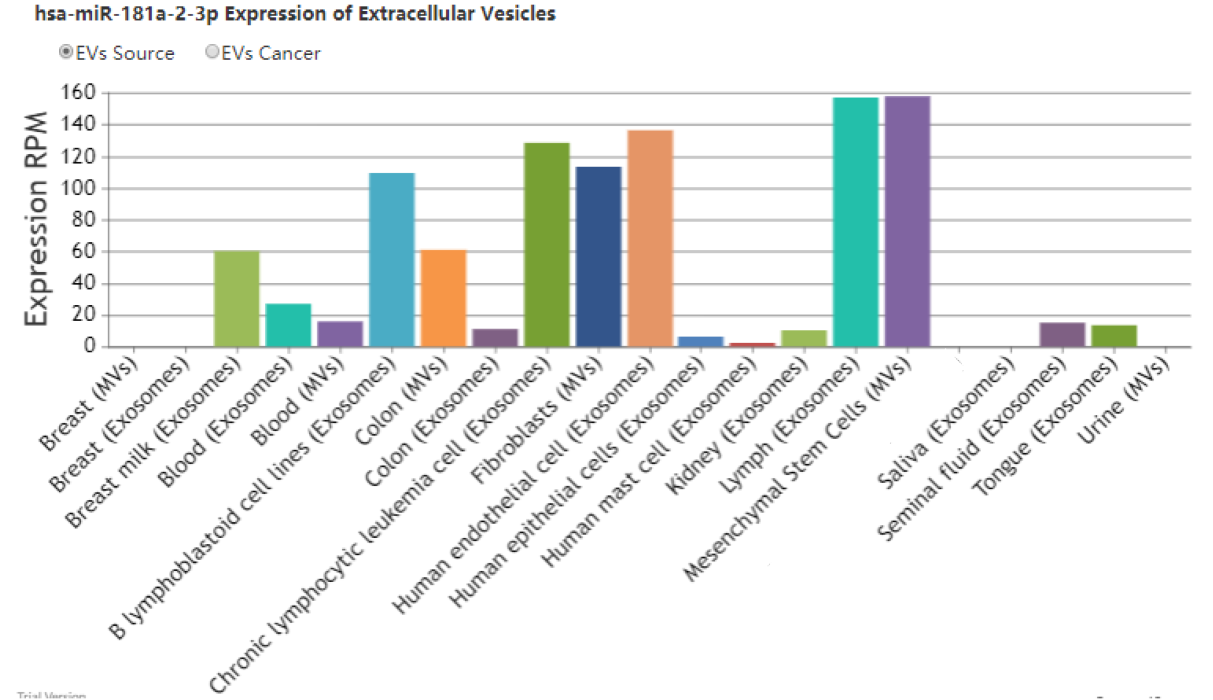


**Supplementary Fig. 2.** MSC-EVs carrying miR-181a-2-3p inhibit 6-OHDA-induced apoptosis and OS of mouse primary neuron cells. **A**, miR-181a-2-3p expression in mouse primary neuron cells detected using RT-qPCR, * *p* < 0.05 *vs.* MSCs-EV-NC-mimic. **B**, Cell proliferation in mouse primary neuron cells. **C**, Apoptosis of mouse primary neuron cells. **D**, SOD level of mouse primary neuron cells detected using SOD kit. **E**, MDA level of mouse primary neuron cells detected using MDA kit. **F**, ROS levels of mouse primary neuron cells detected using DCFH-DA fluorescent staining. B, D, E, F, * *p* < 0.05 *vs.* Blank, # *p* < 0.05 *vs.* 6-OHDA-induced mouse primary neuron cells; $ *p* < 0.05 *vs.* mouse primary neuron cells treated with 6-OHDA + MSCs-EV-NC-mimic.


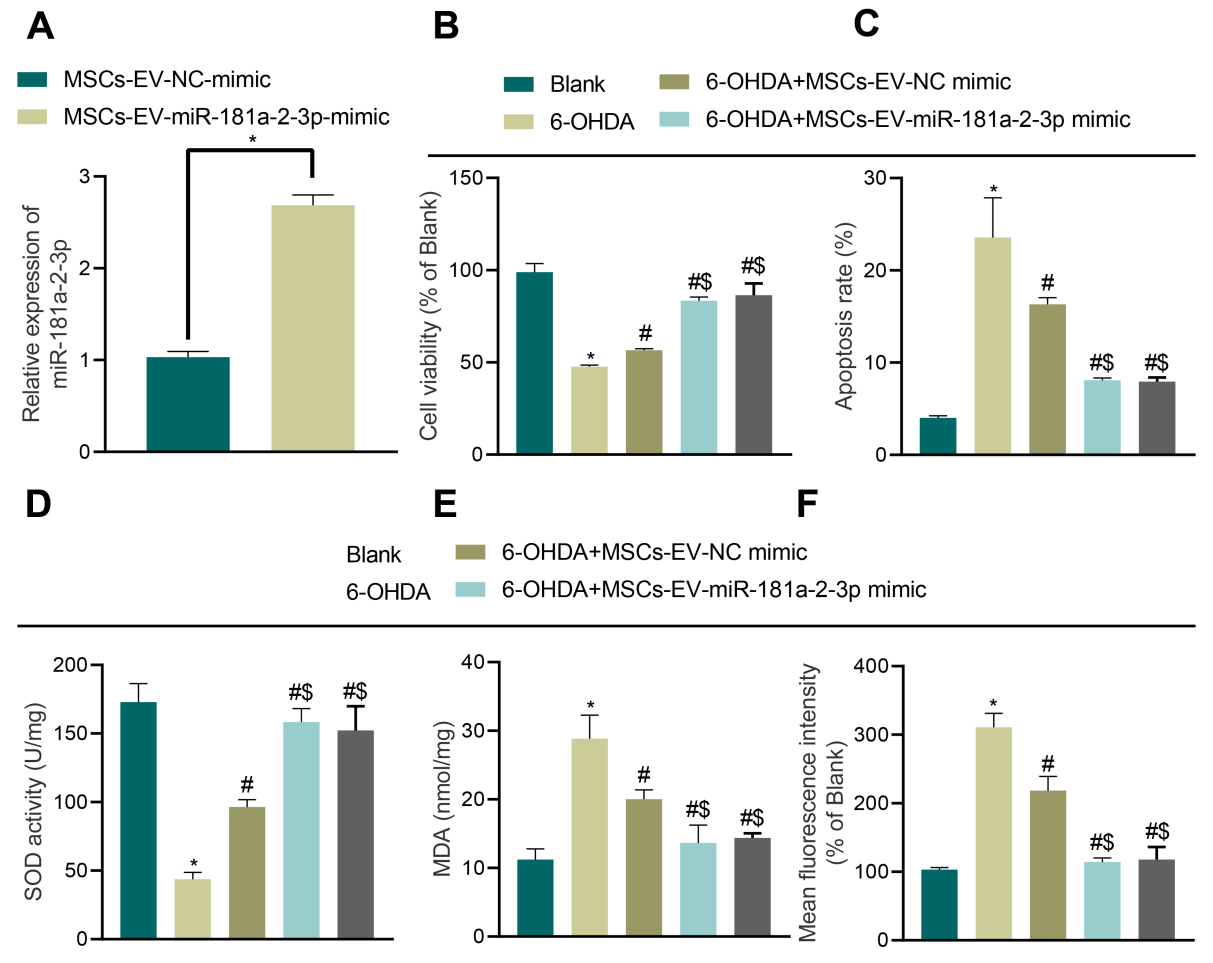


**Supplementary Fig. 3.** Representative images of Western blot and immunohistochemical staining. **A**, Representative images of Western blots of EGR1 protein in SH-SY5Y cells treated with 6-OHDA. **B**, Representative images of Western blots of EGR1 protein in SH-SY5Y cells treated with miR-181a-2-3p mimic or inhibitor. **C**, Representative images of Western blots of EGR1, NOX4, p-p38, and p38 proteins in mouse SN tissues. **D**, TH protein in mouse SN tissues shown by immunohistochemical staining. **E**, Representative images of Western blots of TH protein in mouse SN tissues. **F**, Representative images of Western blots of OS marker 4-HNE in mouse SN tissues. * *p* < 0.05 *vs.* sham-operated mice, # *p* < 0.05*vs.* saline; $ *p* < 0.05 *vs.* MSC-EV-NC-mimic.


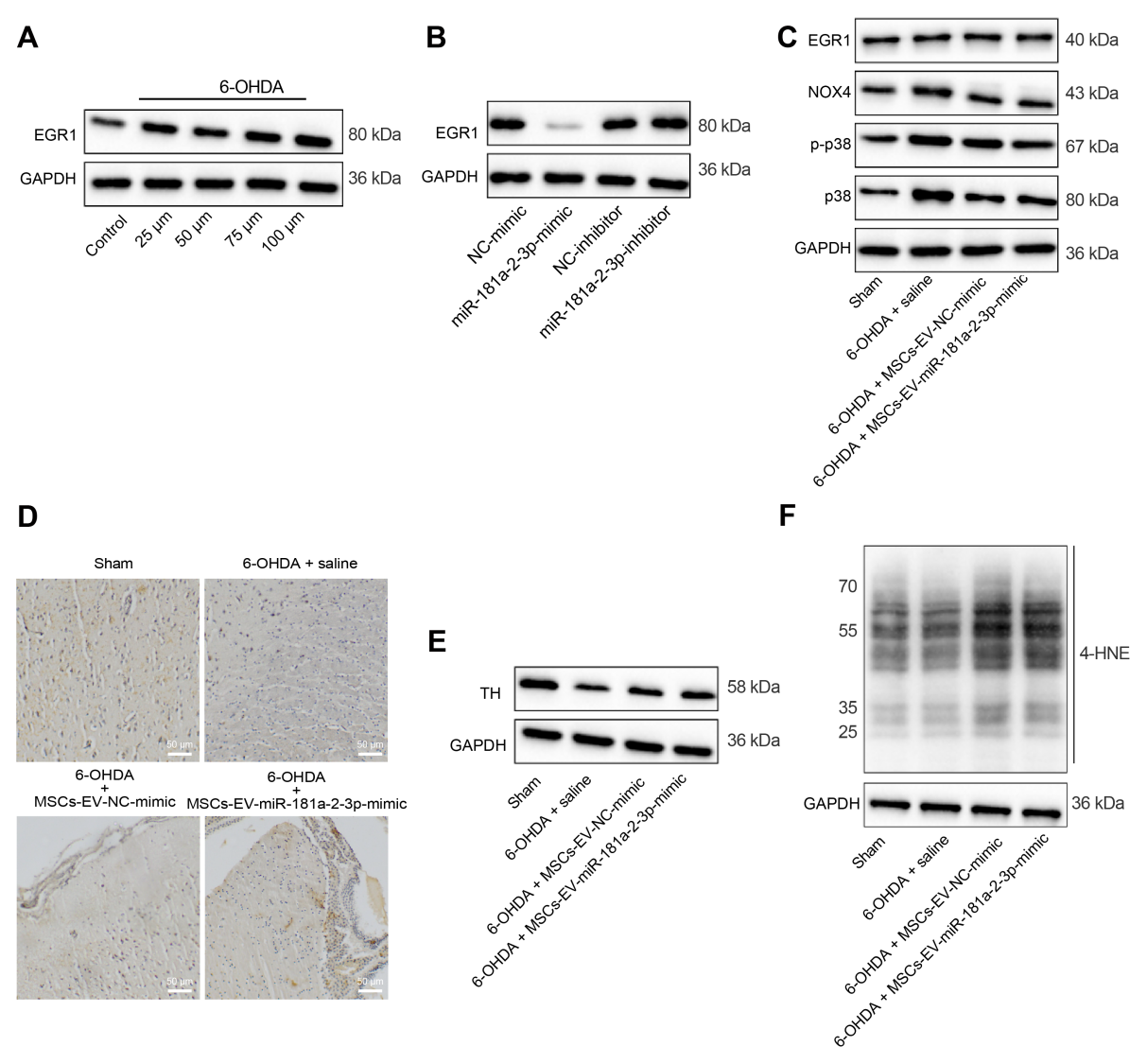

Supplement: Supplementary file 1 — Supplementary Figures [file 41420_2022_823_MOESM1_ESM.docx]
