# Supplementary material for: MicroRNA-181a–2–3p shuttled by mesenchymal stem cell-secreted extracellular vesicles inhibits oxidative stress in Parkinson’s disease by inhibiting EGR1 and NOX4
Source: Cell Death Discov. 2022 Jan 24;8:33. doi: 10.1038/s41420-022-00823-x (PMC8786891; doi:10.1038/s41420-022-00823-x)
Supplement: Supplementary file 2 — Supplementary Tables [file 41420_2022_823_MOESM2_ESM.docx]

**Supplementary Table 1.** RT-qPCR primer sequences

| Targets | Sequences |
| --- | --- |
| miR-181a-2-3p (Homo sapiens) | F: 5-GCGCGACCACGACCGG-3 |
|  | R: 5-AGTGCAGGGTCCGAGGTATT-3 |
| syn-cel-miR-39 | F: 5-GGGGAGCTGATTTCGTCTTG-3 |
|  | R: 5-CTCAACTGGTGTCGTGGAGT-3 |
| EGR1 (Homo sapiens) | F: 5-CACCCAACAGTGGCAACACC-3 |
|  | R: 5-CTGCTGTCGTTGGATGGCAC-3 |
| NOX4 (Homo sapiens) | F: 5-CCGAACACTCTTGGCTTACC-3 |
|  | R: 5-CACTGAGAAGTTGAGGGCATT-3 |
| GAPDH (Homo sapiens) | F: 5-GCACCGTCAAGGCTGAGAAC-3 |
|  | R: 5-TGGTGAAGACGCCAGTGGA-3 |
| U6 (Homo sapiens) | F: 5-CTCGCTTCGGCAGCACA-3 |
|  | R: 5-AACGCTTCACGAATTTGCGT-3 |
| miR-181a-2-3p | F: 5-CGCGCGACCGACCGGA-3 |
|  | R: 5-AGTGCAGGGTCCGAGGTATT-3 |
| U6 (Mus musculus) | F: 5-GCATGACGTCTGCTTTGGA-3 |
|  | R: 5-CCACAATCATTCTGCCATCA-3 |

**Note:** RT-qPCR, reverse transcription quantitative polymerase chain reaction; miR-181a-2-3p, microRNA-R-181a-2-3p; EGR1, early growth response-1; NOX4, NADPH oxidase 4; GAPDH, glyceraldehyde-3-phosphate dehydrogenase.

**Supplementary Table 2.** RT-qPCR primer sequences

| Genes | Sequences |
| --- | --- |
| NOX4 promoter | F: 5-ATCTGGAGGCTCTGCTGGTA-3 |
|  | R: 5-GGCATGCTGTGAGAAGTTCA-3 |

**Note:** RT-qPCR, reverse transcription quantitative polymerase chain reaction; NOX4, NADPH oxidase 4.
